# Mortality Trend of Severe COVID-19 in Under-Vaccinated Population Admitted to ICU in French Amazonia

**DOI:** 10.3390/tropicalmed9010015

**Published:** 2024-01-05

**Authors:** Séverine Matheus, Stéphanie Houcke, Guy Roger Lontsi Ngoulla, Nicolas Higel, Abesetou Ba, Fabrice Cook, Cyrille Gourjault, Flaubert Nkontcho, Magalie Demar, Mathieu Nacher, Félix Djossou, Didier Hommel, Dabor Résiere, Jean Marc Pujo, Hatem Kallel

**Affiliations:** 1 Intensive Care Unit, Cayenne General Hospital, Cayenne 97300, French Guiana; severine.matheus@pasteur.fr (S.M.); stephanie.houcke@ch-cayenne.fr (S.H.); guy.lontsingoulla@ch-cayenne.fr (G.R.L.N.); abesetou.ba@ch-cayenne.fr (A.B.); fabrice.cook@chsf.fr (F.C.); didier.hommel@ch-cayenne.fr (D.H.); 2 Pharmacy Department, Cayenne General Hospital, Cayenne 97300, French Guiana; flaubert.nkontcho@ch-cayenne.fr; 3 Polyvalent Biology Department, Cayenne General Hospital, Cayenne 97300, French Guiana; magalie.demar@ch-cayenne.fr; 4 Tropical Biome and Immunopathology CNRS UMR-9017, Inserm U 1019, Université de Guyane, Cayenne 97300, French Guiana; felix.djossou@ch-cayenne.fr (F.D.); jean.pujo@ch-cayenne.fr (J.M.P.); 5 Clinical Investigation Center Antilles French Guiana (CIC INSERM 1424), Cayenne General Hospital, Cayenne 97300, French Guiana; mathieu.nacher@ch-cayenne.fr; 6 Tropical and Infectious Diseases Department, Cayenne General Hospital, Cayenne 97300, French Guiana; 7 Intensive Care Unit, Martinique University Hospital, Fort de France 97261, Martinique; dabor.resiere@chu-martinique.fr; 8 Emergency Department, Cayenne General Hospital, Cayenne 97300, French Guiana

**Keywords:** COVID-19, Delta variant, Gamma variant, mortality, French Guiana

## Abstract

(1) Background: Until December 2021, French Guiana (FG), located in South America, faced four consecutive COVID-19 epidemic waves. This study sought to analyze the mortality trend of severe COVID-19 patients admitted to the referral ICU of FG. (2) Methods: We conducted a prospective, observational, and non-interventional study in ICU at Cayenne Hospital. We included 383 patients older than 18 admitted with SARS-CoV-2-related pneumonia hospitalized from May 2020 to December 2021. The study covers three periods. Period 1 (Waves 1 and 2, original variant), period 2 (Wave 3, Gamma variant), and period 3 (Wave 4, Delta variant). (3) Results: The median age was 63 years (52–70). Frailty was diagnosed in 36 patients over 70 (32.4%). Only 4.8% of patients were vaccinated. The median ICU LOS was 10 days (6–19). Hospital mortality was 37.3%. It was 30.9% in period 1, 36.6% in period 2 (*p* = 0.329 vs. period 1), and 47.1% in period 3 (0.015 vs. period 1). In multivariate analysis, independent factors associated with hospital mortality included age greater than 40 years (]40–60 years] OR = 5.2, 95%CI: 1.4–19.5; (]60–70 years] OR = 8.5, 95%CI: 2.2–32; (]70+ years] OR = 17.9, 95%CI: 4.5–70.9), frailty (OR = 5.6, 95%CI: 2.2–17.2), immunosuppression (OR = 2.6, 95%CI: 1.05–6.7), and MV use (OR = 11, 95%CI: 6.1–19.9). This model had an overall sensitivity of 72%, a specificity of 80.4%, a positive predictive value of 68.7%, and a negative predictive value of 82.8%. (4) Conclusions: The mortality of severe COVID-19 patients in French Amazonia was higher during the Delta variant wave. This over-death could be explained by the virulence of the responsible SARS-CoV-2 variant and the under-vaccination coverage of the studied population.

## 1. Introduction

The first COVID-19 epidemic wave was declared in French Guiana (FG) in South America in May 2020. Until December 2021, FG faced four waves of SARS-CoV-2 epidemic. The first wave was delayed from that encountered in mainland France, and its peak was reached gradually within five weeks. This allowed for an increase in the healthcare system preparedness, a surge in the intensive care bed capacity, and training medical and non-medical teams. It also allowed the benefit of national solidarity aid regarding equipment and human resources.

SARS-CoV-2 waves in FG were declared when the incidence density in the community exceeded a threshold of 150 infected cases/100,000 inhabitants or when the dominant variant changed [1]. Accordingly, patients’ characteristics and outcomes can vary from wave to wave. Many studies have described the characteristics of patients with COVID-19 [2,3,4]. They have investigated outcomes, especially among older and vulnerable populations [5,6]. In some studies, the mortality rate reached 60% [7,8] and decreased over time, mainly because of vaccination coverage [9,10]. The main factors associated with increased mortality were age > 55, pre-existing comorbidities, acute respiratory distress syndrome, extensive lung involvement in computed tomography findings, and organ failure [9,11].

Complete vaccination for SARS-CoV-2 showed protective effectiveness in saving lives, mainly when the vaccine coverage rate exceeds 60% [12,13]. Tenforde et al., in a large case–control study of adults hospitalized for COVID-19, showed that complete vaccination reduces progression to death or invasive mechanical ventilation [14]. In FG, the Pfizer–BioNTech COVID-19 vaccine has been rolled out for free by the local sanitary authorities since January 2021. Unfortunately, there was a high rate of vaccine hesitancy with a low coverage rate in the local population. This may have driven a higher rate of hospitalizations, ICU admissions, and over-deaths [15].

The primary objective of this study was to describe the mortality trend related to severe COVID-19 in an under-vaccinated population in French Amazonia. The secondary objective was to investigate factors associated with mortality in these patients.

## 2. Materials and Methods

Our study is prospective, observational, and non-interventional. It was conducted from 1 May 2020 to 31 December 2021, in the ICU at Cayenne General Hospital. We included all patients older than 18 admitted with COVID-19 pneumonia. We excluded patients with positive SARS-CoV-2 screening without respiratory symptoms. Only the first ICU admission during the same hospital stay was considered. 

Our unit is the referral ICU in FG [16]. It works per European and French standards. The initial ICU capacity accounted for 11 beds. It was increased to 41 beds during the crisis with a consequent surge in human resources and equipment needs.

The management protocol of severe COVID-19 pneumonia associated High Flow Nasal Cannula Oxygen (HFNCO), non-invasive mechanical ventilation (NIV), invasive mechanical ventilation (MV), and prone position in sedated and non-sedated patients. We used therapeutic anticoagulation with Heparin (target anti-Xa at 0.4–0.6 UI/mL). This regimen was changed in September 2021 to pharmacologic thromboprophylaxis [17]. Dexamethasone was used with an initial protocol similar to Villar et al. (20 mg daily for 5 days followed by 10 mg daily for 5 days) [18] and changed to 6 mg daily for 10 days in August 2020 [19]. Systematic antimicrobial therapy by cefotaxime alone or in combination with levofloxacin was prescribed at admission to ICU till September 2020. Antibiotics were then reserved only for documented infections.

Data were collected in a datasheet, and patients were referred by numbers to grant privacy protection. The following parameters were collected: gender, age, BMI score, simplified acute physiology score (SAPS II) [20], organ failure [21], comorbidities, frailty [22], management strategy (respiratory support, vasopressors, renal replacement therapy, etc.), and outcome (ICU and hospital length of stay (LOS) and mortality).

The waves’ dates were defined by the local sanitary authority according to epidemiological data on the responsible strain [1]. In our study, we classified the four epidemic waves in three periods as follows:

- Period 1 (Waves 1 and 2): from May to September 2020, and from November 2020 to February 2021 caused by the original SARS-CoV-2 strain.
- Period 2 (Wave 3): from March to July 2021, caused by Gamma (P.1) and Alpha (B.1.1.7) variants circulation (88% and 12%, respectively).
- Period 3 (Wave 4): from August to December 2021, caused by Delta (B.1.617) and Gamma (P.1) variants circulation (78% and 21%, respectively).

We calculated the weekly occupancy rates (W_OR_), defined as the number of hospitalized patients (COVID and non-COVID) divided by the ICU bed capacity during the considered week (cumulative number of daily open beds during the week). Occupancy rate per period was calculated as the mean W_OR_ during the studied period. 

Results are reported as number of patients in whom the data were recorded (Nb), median and inter-quartile range (IQR: 1st–3rd quartiles), or numbers with percentages. Initial bivariate statistical comparisons for categorical variables were conducted using the Chi-square or Fisher’s exact test. Continuous variables were compared using the Mann–Whitney U-test. We used multivariable logistic regression to identify patients’ characteristics associated with hospital death. Non-redundant variables selected via bivariate analysis (*p* ≤ 0.05) and considered clinically relevant were entered into the logistic regression model. Postestimation commands allowed the sensitivity and specificity of the model and the area under the curve to be obtained. Kaplan–Meier analysis was used to estimate the survival function. Results are expressed as odds ratios (OR) with 95% confidence intervals (95%CI). Statistical tests were two-tailed, and *p* ≤ 0.05 was considered significant.

Statistical analyses were carried out with Excel (2010 Microsoft Corporation, Redmond, DC, USA) and IBM SPSS Statistics for Windows, version 24 (IBM Corp., Armonk, NY, USA), and STATA (STATA Corp., College Station, TX, USA).

## 3. Results

**Figure 1:**
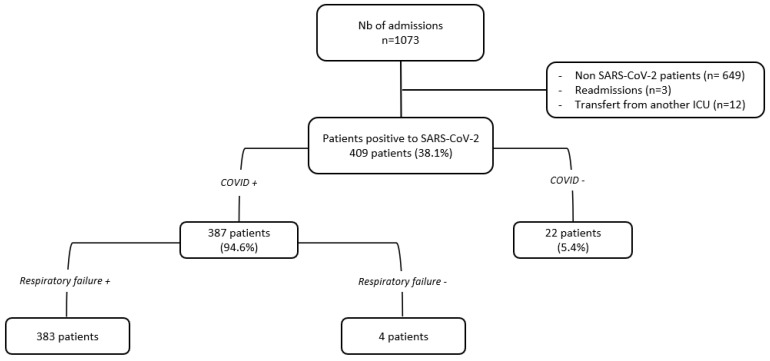
The flow-chart of the study.

**Figure 2:**
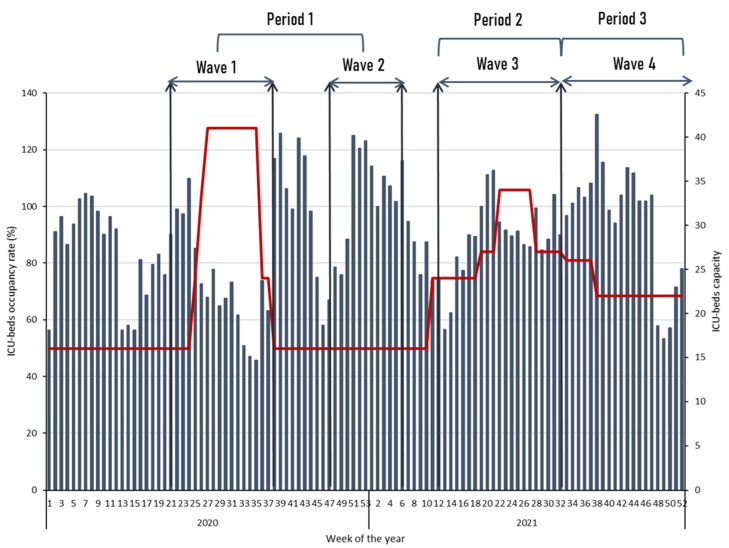
ICU-bed capacity (in red line) and occupancy rate (in bars) during the study period.

During the study period, 1073 patients were admitted to ICU. SARS-CoV-2 screening was positive in 409 patients, and 383 suffered from COVID-19 pneumonia (Figure 1). The median W_OR_ was 90.6% (IQR: 74.3–104.7) (Figure 2). It was 85.1% (IQR: 67.6–109.8) in period 1, 89.5% (IQR: 83.4–97) in period 2 (*p* = 0.723 compared to period 1), and 101.9% (IQR: 81.9–107.8) in period 3 (*p* = 0.247 compared to period 1).

### 3.1. Demographic Characteristics and Comorbidities

**Figure 3:**
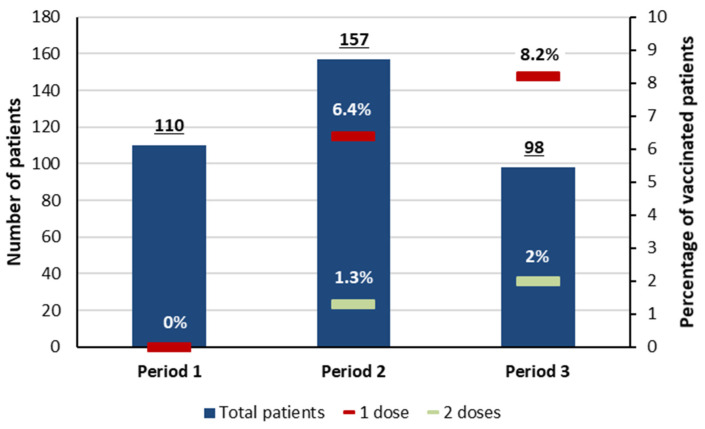
The vaccination rate during the study periods. In bar: number of patients in whom the vaccination status was available. Red mark: the vaccination rate (at least one dose). Green mark: the vaccination rate (two doses).

The number of patients hospitalized for COVID-19 pneumonia was 110 during the first period, 161 during the second, and 104 during the third period. The vaccination rate (at least one dose) was 4.8% (18/373) and a complete vaccination (two doses) was registered in only four patients. It was zero during period 1, 6.4% (10/157) during period 2, and 8.2% (8/98) during period 3 (Figure 3). Overall (383 patients), the median age was 63 years (IQR: 52–70), and 208 patients (54.3%) were male. Patients were aged more than 70 in 111 cases (29%). The median age was 63 years (IQR: 53–70) in men and 54 (IQR: 54–73) in women (*p* = 0.223). BMI was 30 kg/m^2^ (IQR: 26–33) in men and 32 (IQR: 28–36) in women (*p* = 0.043). Frailty was diagnosed in 36 patients aged more than 70 (32.4%) and in 6 patients (4.8%) aged 60 to 69. The most registered comorbidities were arterial hypertension (61.4%), obesity (49.1%), and diabetes (42.3%). These comorbidities were concomitant in 75 patients (19.6%). The time between the onset of symptoms and hospitalization was 7 days (IQR: 4–9). At admission to ICU, 179 patients (46.7%) received HFNCO, 102 (26.6%) received HFNCO + NIV, 6 (1.5%) received NIV, and 96 (25.1%) received MV. The maximal respiratory support used during ICU stay was MV in 48.3% (+92.7%) and HFNCO in 36% of cases (−22.9%) (Table S1). 

The analysis of period two compared to period one showed a lower SAPS II, a lower rate of male gender, diabetes mellitus, MV use, and RRT use; and a higher rate of NIV use. The analysis of period three compared to period one showed a lower rate of male gender, MV use, and RRT use; and a higher rate of NIV use. The time from admission to MV was longer in period 3 (Table S1). 

### 3.2. Outcome

ICU LOS was 10 days (IQR: 6–19). It was 9 days (IQR: 6–15) in survivors and 14 (IQR: 7–23) in non-survivors (*p* = 0.003). It was equal to or higher than 30 days in 47 cases (12.3%). Hospital LOS was 18 days (IQR: 12–29). It was higher in survivors (17 vs. 18, *p* = 0.010). Deceased patients were more severe at admission (SAPS II at 33 vs. 29, *p* < 0.001) and more likely to receive MV (93.6%, *p* = 0.003) (Table S2). ICU and hospital LOS were lower in period 2. Hospital mortality was higher in period 2 but without a statistical difference (30.9% vs. 36.6% in period 1, *p* = 0.329). The ICU and hospital LOS were lower, and the hospital mortality rate was higher during period 3 (30.9% vs. 47.1% in period 1, *p* = 0.015) (Table S3).

**Figure 4:**
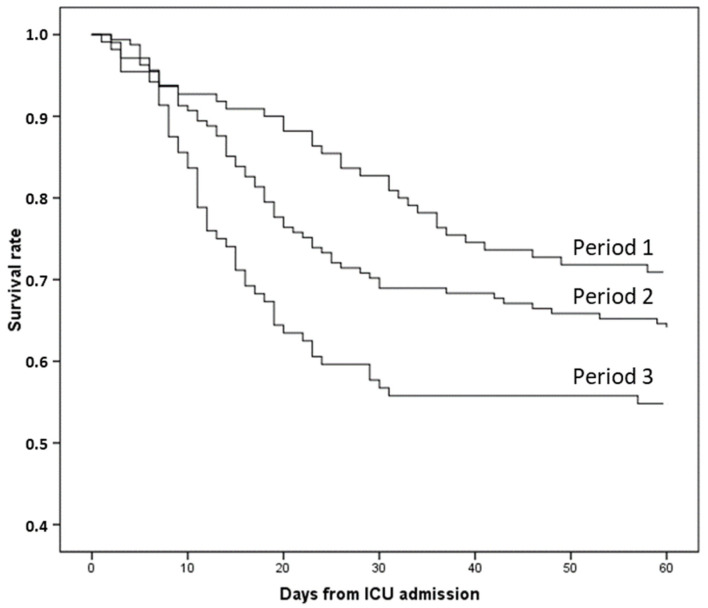
Sixty-day mortality according to the period of analysis. Period 1 vs. period 2 (Log Rank = 0.176), Period 1 vs. period 3 (Log Rank = 0.005).

**Figure 5:**
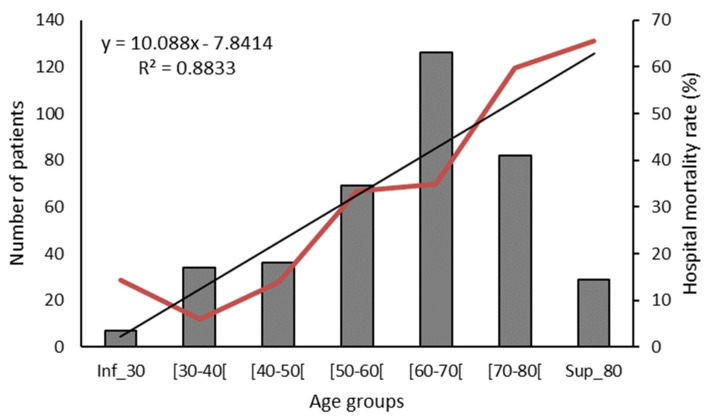
Hospital mortality according to age groups (r = 0.939, *p* < 0.001). Bars indicate the number of patients in each age group, the red line represents the hospital mortality rate, and the black line represents the tendency line of hospital mortality according to age groups.

The ICU mortality rate was 36% and the hospital mortality was 37.3%. The causes of death were septic shock in 67 cases (46.9%), refractory hypoxemia in 53 cases (37.1%), probable pulmonary embolism in 11 cases (7.7%), myocardial infarction in 6 cases (4.2%), cardiogenic shock in 2 cases (1.4%), hemorrhagic shock in 2 cases (1.4%), hemorrhagic stroke in 1 case, and decompensated liver cirrhosis in 1 case. In 54 cases (37.8%) the decision to withhold or withdraw life-sustaining support was made. Hospital mortality in frail patients was 71.4%. Hospital mortality was 30.9% in period 1, 36.6% in period 2 (*p* = 0.329 vs. period 1), and 47.1% in period 3 (*p* = 0.015 vs. period 1) (Figure 4). There was a significant relationship between hospital mortality and patients’ age (r^2^ = 0.883, *p* < 0.001) (Figure 5). ICU and hospital LOS were more prolonged, and hospital mortality was higher in patients receiving MV regardless of the study period (Table 1).

Factors associated with hospital mortality in univariate analysis are reported in Table S2. The main parameters were age, frailty, severity at admission (SAPS II), comorbidities (hypertension, diabetes, chronic renal failure, and immunosuppression), organ failure at ICU admission (hemodynamic, neurologic, and renal), and MV use. Multivariate analysis including these variables in the model found that older age groups were at much greater risk than those aged 40 years or less (]40–60 years] OR = 5.2, 95%CI: 1.4–19.5, *p* = <0.001; (]60–70 years] OR = 8.5 95%CI: 2.2–32, *p* = 0.002; (]70+ years] OR = 17.9, 95%CI: 4.5–70.9, *p* < 0.001), frailty (OR = 5.6, 95%CI: 2.2–17.2, *p* < 0.001), immunosuppression (OR = 2.6, 95%CI: 1.05–6.7, *p* = 0.04), and MV use (OR = 11, 95%CI: 6.1–19.9, *p* < 0.001) as factors independently associated with hospital mortality. The above model had an overall sensitivity of 72%, a specificity of 80.4%, a positive predictive value of 68.7%, and a negative predictive value of 82.8%. The area under the ROC curve was 0.843.

## 4. Discussion

Our study describes the epidemiology and mortality trend of severe COVID-19 patients admitted to the ICU during four epidemic waves in an under-vaccinated population in French Amazonia. It shows that the mortality rate increased from wave to wave. Age, frailty, immunosuppression, and MV use are independently associated with hospital mortality. 

The rapid progression of COVID-19 has put hospitals under significant pressure. To deal with the crisis, emergency and intensive care physicians were forced to select patients, a process called “triage” [23,24]. Patients were categorized based on age and previous physiological status [22]. Overall, patients’ admission to ICU and outcomes were influenced by the severity of the disease, comorbidities, and ICU bed availability. In FG, due to the epidemic spread, we increased the ICU bed capacity, and we created new ICUs in the other two hospitals in the department called “ephemeral ICU beds”. As a result, there was no need for “triage” in our hospital. However, the average occupancy rate was 90.6%, without a difference between the periods. In this context, it is well described that surging ICU during COVID-19 pandemic can be associated with a lower quality of care and a greater risk of death [25].

Several studies investigated mortality in COVID-19 patients at 28 [19], 30 [26,27], and 90 days [9,28] from ICU admission, while others have focused on ICU and hospital mortality [27,29]. In our study, we assessed the hospital mortality of severe COVID-19 patients. Indeed, 45 patients (11.7%) were hospitalized in ICU for more than 30 days, and 22 deceased patients were still alive on day 30. In addition, patients hospitalized for more than 30 days were the most severe at admission and were most often under MV. Accordingly, assessing mortality at 30 days from ICU admission can underestimate the potentially unfavorable outcome. 

At the beginning of the COVID-19 crisis, the mortality rate was as high as 60% [7,8] and reached 70.3% in patients receiving MV [30]. Some studies have reported a decreased mortality trend over time [9,10,31,32,33], whereas others found that mortality did not differ between waves [29]. Overall, the reported mortality rate in large trials was about 30%. Indeed, in a multicenter Spanish study, Carbonell et al. [30] found 30.7% overall mortality in ICU without difference between COVID-19 waves. Nevertheless, they found a significant reduction in the ICU and hospital LOS in survivors during the second/third waves, which they explained by the change of the management protocol over time and that physicians became more familiar with the disease. In the COVID-ICU group study [9], the 90-day mortality was 31% and decreased from 42 to 25% over the study period. In a Dutch study [10], the crude hospital mortality was 29.9% in wave 1, 32.0% in wave 2, and it decreased to 23.4% in wave 3. This study found fewer patients treated with MV and vasopressors during wave 3. Accordingly, the authors cannot exclude that patients’ triage might have influenced their results. In a Brazilian study [34], the mortality rate in ICU was 57%. It increased by 3.3% per day during ICU stay. Finally, Serafim et al. [35], in a review including 69,093 patients, found an overall ICU mortality rate of 32.3%. More than half the patients admitted to ICU required MV (58%) and had a mortality rate of 59%. In our study, the overall ICU mortality rate was 36%. It was, respectively 30, 34.2, and 47.1% during periods 1, 2, and 3, and hospital mortality was within similar ranges. The rates recorded in periods 1 and 2 were similar to those reported in the literature. Meanwhile, the mortality recorded in period three was higher without significant difference in patients’ characteristics. However, in period 3, the time between the first symptoms and hospitalization, NIV use, and time from ICU admission to MV were higher than in period 1. On the other hand, patients in period 3 were less exposed to antibiotics prior to MV, and had a lower need for RRT. These differences are probably related to the involved virus variant and the subsequent characteristics of the disease. Overall, one can suppose that the higher mortality rate observed in period 3 could be explained by the responsible SARS-CoV-2 variant. Nevertheless, we cannot exclude the impact of changes in the management strategy on outcome.

Several studies have focused on factors associated with mortality in COVID patients. They include age, frailty, ventilatory support, time taken to introduce MV, the causal virus variant, and vaccination coverage [36,37,38,39]. Wendel-Garcia et al. found that HFNC use was associated with lower ICU mortality, while NIV was associated with higher ICU mortality [36]. Camous et al., in a large case series from Guadeloupe, showed that very late MV, defined as intubation after day 7 of dexamethasone therapy, was associated with a high mortality rate of 87% [37]. Manrique et al. reported that implementing an early intubation approach is an independent protective factor for mortality [38]. In this study, late intubation was defined as intubation > 24 h after ICU admission, and the severity of the disease was lower in the late intubation group. Our study is prospective observational and non-interventional. Intubation and respiratory support were left to the discretion of attending physicians. We found that NIV use was higher in periods 2 and 3 than in period 1. Mechanical ventilation use was higher in period 1 than in periods 2 and 3. Also, the time from ICU admission to MV was higher in period 3 than periods 1 and 2. However, NIV use was not statistically different between survivors and non-survivors, while MV use and the time from ICU admission to MV were higher in non-survivors. Accordingly, we can suggest that these two parameters may have influenced the outcome of our patients.

Several studies focused on elderly and vulnerable patients, whose predicted outcome is poor when MV is needed. These patients were recused from critical care and MV, especially in case of ICU bed shortage. The clinical frailty score was used mainly in patients older than 70 [6,28] and sometimes in those over 65 years [22,40]. In patients over 70 years with COVID-19 admitted to the ICU, the 90-day mortality was 46% and reached 67% among patients over 80 [28]. In the COVIP study [6], the mortality rate was 34% in fit, 47% in vulnerable, and 59% in frail patients, without differences in 30-day survival between different age categories in frail patients. After controlling for confounding variables, frailty was independently associated with lower survival. In our study, age was an independent risk factor for mortality. Frailty was diagnosed in 11% of patients. Mortality in frail patients was 71.4%. Our results align with the literature and sustain the use of age and frailty in selecting patients for ICU admission, mainly in case of ICU bed shortage.

In our study, the dominant variants were Gamma during period 2 and Delta during period 3. Gamma variant emerged in Brazil in June 2020 [41] and reached FG because of the geographical proximity. Interestingly, FG is the sole French territory where the Gamma variant was registered. Delta variant emerged in India in late 2020. It showed a 40–60% increase in transmissibility compared to the Alpha variant, which was twice as contagious as the original strain [42]. In a large Canadian study, ICU admission and death probability were 1.9- and 1.33-fold higher with the Delta variant than non-variants of interest [43]. Additionally, unvaccinated patients faced 11 times greater risk of death from the Delta variant compared to vaccinated people [44]. Tabatabai et al. showed that the odds of death were 3.45-fold higher during Delta than Alpha periods [45]. In a large study in the United States, Liu et al. showed a 73.8% decrease in the case-fatality rate related to the Delta variant in fully vaccinated people compared to unvaccinated people [46]. In our study, the ICU and hospital mortality were higher during the third period, in which 84.8% of patients under MV died. These findings could be explained by the dominant circulating strain (80% of screened variants were Delta) and the under-vaccination coverage (8.2% of patients received one vaccine dose and only four patients were completely vaccinated). Indeed, with the same vaccination rates as mainland France, Nacher et al. estimated that 62.4% of ICU admissions would have been avoided in FG [15]. This result corroborates a large French study showing that vaccination is associated with a lower risk of invasive MV and in-hospital death [39].

This study has potential limitations. First, this is a monocentric study. However, our unit accounted for 76% of ICU beds in FG and most severe patients were transferred to our unit. In addition, ICU hospitalizations and death were monitored by the local French sanitary authorities. Accordingly, 80% of severe COVID-19 patients in FG were managed in our unit. To this end, our study accurately shows what happened in FG during the COVID-19 crisis. Second, there were changes in the management protocol during the study period. These changes followed the scientific updates during the crisis and were observed in all studies dealing with COVID-19 [19,29,30]. Third, we separate three different periods according to the dominant circulating virus variant. Indeed, in period 1 there were two distinct waves with changes in the protocol management. A subgroup analysis comparing wave 1 to wave 2 did not show differences in patients’ epidemiological characteristics and outcomes. However, this study describes the epidemiology and outcome of severe COVID-19 patients admitted to ICU in a high-income context (French department) with a middle- to low-income and under-vaccinated population in South America.

## 5. Conclusions

Our study shows that the mortality of severe COVID-19 patients was higher during the Delta variant wave in French Amazonia, with a shorter period from the onset of symptoms to ICU admission. This over-death could be explained by the virulence of the responsible SARS-CoV-2 variant and the under-vaccination coverage of the studied population without excluding the impact of changes in the management strategy on outcome.

## Figures and Tables

**Table 1:** Hospital mortality, ICU, and Hospital LOS according to the period of the study and the maximal respiratory support.

Maximal Respiratory Support	Overall (*n* = 383)	Period 1 (*n* = 110)	Period 2 (*n* = 161)	*p* *	Period 3 (*n* = 104)	*p* ^$^
High-flow nasal cannula oxygen	*n* = 138 (36.0%)	*n* = 39 (35.5%)	*n* = 61 (37.9%)	0.683	*n* = 34 (32.7%)	0.670
Hospital mortality	18 (13%)	2 (5.1%)	10 (16.4%)	0.091	5 (14.7%)	0.166
ICU LOS (days)	7 (5–9)	8 (6–12)	6 (4–8)	0.004	6 (5–10)	0.095
Hospital LOS (days)	16 (12–20)	19 (16–23)	14 (11–17)	0.000	15 (11–18)	0.011
Non-invasive mechanical ventilation	*n* = 60 (15.7%)	*n* = 4 (3.6%)	*n* = 30 (18.6%)	<0.001	*n* = 24 (23.1%)	<0.001
Hospital mortality	16 (26.7%)	2 (50%)	9 (30%)	0.580	5 (20.8%)	0.212
ICU LOS (days)	10 (7–14)	13 (7–29)	9 (5–10)	0.180	11 (7–15)	0.776
Hospital LOS (days)	17 (12–26)	16 (9–48)	16 (12–25)	0.979	17 (11–23)	0.975
Invasive mechanical ventilation	*n* = 185 (48.3%)	*n* = 67 (60.9%)	*n* = 70 (43.5%)	0.005	*n* = 46 (44.2%)	0.015
Hospital mortality	109 (58.9%)	30 (44.8%)	40 (57.1%)	0.148	39 (84.8%)	<0.001
ICU LOS (days)	18 (10–28)	21 (9–33)	19 (11–28)	0.411	13 (7–25)	0.088
Hospital LOS (days)	23 (13–39)	26 (13–42)	25 (15–41)	0.973	16 (9–29)	0.027

Nb: Number of patients in whom the data were recorded; ICU: intensive care unit; LOS: length of stay. * Period 2 vs. period 1; ^$^ period 3 vs. period 1.

## Data Availability

All data supporting reported results are available from the corresponding author upon request.

## Supplementary Materials

The following supporting information can be downloaded at: https://www.mdpi.com/article/10.3390/tropicalmed9010015/s1, Table S1: Epidemiologic characteristics and management according to the study periods; Table S2: Comparison of survivors and non-survivors at hospital discharge; Table S3: Outcome of patients according to the study periods.
