# Supplementary material for: Mortality Trend of Severe COVID-19 in Under-Vaccinated Population Admitted to ICU in French Amazonia
Source: Trop Med Infect Dis. 2024 Jan 5;9(1):15. doi: 10.3390/tropicalmed9010015 (PMC10820344; doi:10.3390/tropicalmed9010015)
Supplement: Supplementary file 1 [file tropicalmed-09-00015-s001.zip › tropicalmed-2786498-supplementary.pdf]

Supplemental digital content

Mortality trend of severe COVID-19 in under-vaccinated population admitted to ICU in French Amazonia.

Table of contents:

Table S1: This is a table. Epidemiologic characteristics and management according to the study periods 2

Table S2: This is a table. Comparison of survivors and non-survivors at hospital discharge 5

Table S3: This is a table. Outcome of patients according to the study periods 7

Table S1: This is a table. Epidemiologic characteristics and management according to the study periods

	Total population		Period 1		Period 2			Period 3		
**Parameter**	**Nb**	**Result**	**Nb**	**Result**	**Nb**	**Result**	**p***	**Nb**	**Result**	**p$**
Age (years)	383	63 (54 - 71)	110	64 (52 - 70)	161	62 (54 - 71)	0.890	104	65 (55 - 72)	0.368
Male gender	383	208 (54.3%)	110	72 (65.5%)	161	86 (53.4%)	0.048	104	45 (43.3%)	0.001
BMI (Kg/m^2)	345	30 (27 - 35)	100	30 (27 - 33)	144	30 (26 - 35)	0.265	93	30 (27 - 34)	0.191
Frailty	383	42 (11%)	110	8 (7.3%)	161	21 (13%)	0.131	104	12 (11.5%)	0.284
Simplified Acute Physiology Score	381	29 (24 - 37)	110	32 (26 - 42)	160	29 (23 - 35)	0.009	103	30 (24 - 37)	0.103
**Comorbidities**										
Diabetes mellitus	383	162 (42.3%)	110	58 (52.7%)	161	61 (37.9%)	0.016	104	41 (39.4%)	0.051
Hypertension	383	235 (61.4%)	110	67 (60.9%)	161	88 (54.7%)	0.307	104	75 (72.1%)	0.083
Chronic respiratory failure	383	28 (7.3%)	110	8 (7.3%)	161	15 (9.3%)	0.553	104	4 (3.8%)	0.276
Chronic renal failure	383	42 (11%)	110	16 (14.5%)	161	11 (6.8%)	0.037	104	14 (13.5%)	0.819
Dialysis	383	14 (3.7%)	110	8 (7.3%)	161	4 (2.5%)	0.060	104	2 (1.9%)	0.064
Renal transplantation	383	4 (1%)	110	3 (2.7%)	161	0 (0%)	0.035	104	1 (1%)	0.340
Cardiac failure	383	31 (8.1%)	110	12 (10.9%)	161	13 (8.1%)	0.428	104	6 (5.8%)	0.176
Obesity	383	188 (49.1%)	110	50 (45.5%)	161	85 (52.8%)	0.235	104	49 (47.1%)	0.808
Pregnancy (within women)	175	19 (10.9%)	38	5 (13.2%)	75	8 (10.7%)	0.695	59	6 (10.2%)	0.650
Gestational age (weeks of gestation)	18	30 (28 - 37)	5	28 (28 - 35)	7	29 (28 - 35)	0.934	6	34 (29 - 38)	0.518
Sickle cell disease	383	5 (1.3%)	110	0 (0%)	161	3 (1.9%)	0.150	104	2 (1.9%)	0.144
Malignancy	383	12 (3.1%)	110	3 (2.7%)	161	3 (1.9%)	0.635	104	4 (3.8%)	0.646
Immunosuppression	383	28 (7.3%)	110	8 (7.3%)	161	7 (4.3%)	0.301	104	11 (10.6%)	0.396

Table S1: This is a table. Epidemiologic characteristics and management according to the study periods (Follow)

**Time to hospitalization and ICU admission**										
Time between first symptoms and hospitalization (days)	364	7 (4 - 9)	106	6 (3 - 8)	151	7 (4 - 9)	0.062	99	7 (5 - 9)	0.015
Time between Hospitalization and ICU admission (days)	383	1 (0 - 4)	110	1 (0 - 4)	161	1 (0 - 4)	0.426	104	2 (0 - 3)	0.956
**Clinical manifestations**										
Number of organ failure	383	1 (1 - 1)	110	1 (1 - 2)	161	1 (1 - 1)	0.553	104	1 (1 - 1)	0.413
Hemodynamic failure	383	24 (6.3%)	110	12 (10.9%)	161	8 (5%)	0.066	104	4 (3.8%)	0.050
Respiratory failure	383	383 (100%)	110	110 (100%)	161	161 (100%)	-	104	104 (100%)	-
Neurologic failure	383	25 (6.5%)	110	6 (5.5%)	161	11 (6.8%)	0.646	104	7 (6.7%)	0.676
Hematologic failure	383	7 (1.8%)	110	3 (2.7%)	161	0 (0%)	0.035	104	3 (2.9%)	0.944
Renal failure	383	70 (18.3%)	110	22 (20%)	161	27 (16.8%)	0.498	104	15 (14.4%)	0.281
Liver failure	383	10 (2.6%)	110	2 (1.8%)	161	4 (2.5%)	0.714	104	2 (1.9%)	0.955
Infection at admission	383	8 (2.1%)	110	2 (1.8%)	161	3 (1.9%)	0.978	104	3 (2.9%)	0.606
**Chest Computed-Tomography**	**383**	**347 (90.6%)**	**110**	**92 (83.6%)**	**161**	**150 (93.2%)**	**0.013**	**104**	**97 (93.3%)**	**0.028**
Alveolar condensations	347	120 (34.6%)	92	17 (18.5%)	150	78 (52%)	0.000	97	21 (21.6%)	0.587
Interstitial infiltrates	347	331 (95.4%)	92	91 (98.9%)	150	140 (93.3%)	0.043	97	92 (94.8%)	0.111
Pleural effusion	347	29 (8.4%)	92	6 (6.5%)	150	16 (10.7%)	0.276	97	6 (6.2%)	0.925
Posterior condensations	347	181 (52.2%)	92	28 (30.4%)	150	95 (63.3%)	0.000	97	55 (56.7%)	0.000
Contrast agent administration	347	267 (76.9%)	92	87 (94.6%)	150	119 (79.3%)	0.001	97	53 (54.6%)	0.000
Pulmonary embolism	267	34 (12.7%)	87	9 (10.3%)	119	12 (10.1%)	0.951	53	11 (20.8%)	0.088
Percentage of lesion	341	70 (50 - 75)	89	70 (50 - 75)	149	75 (50 - 75)	0.922	97	50 (50 - 75)	0.068

Table S1: This is a table. Epidemiologic characteristics and management according to the study periods (Follow)

**Respiratory support at ICU admission**										
High-flow nasal cannula oxygen	383	281 (73.4%)	110	69 (62.7%)	161	118 (73.3%)	0.065	104	88 (84.6%)	0.000
Non-invasive mechanical ventilation	383	108 (28.2%)	110	10 (9.1%)	161	45 (28%)	0.000	104	51 (49%)	0.000
Invasive mechanical ventilation	383	96 (25.1%)	110	39 (35.5%)	161	41 (25.5%)	0.077	104	14 (13.5%)	0.000
**Maximal respiratory support during hospitalization**										
High-flow nasal cannula oxygen	383	138 (36%)	110	39 (35.5%)	161	61 (37.9%)	0.683	104	34 (32.7%)	0.670
HFNCO duration (days)	125	6 (4 - 8)	33	6 (5 - 10)	60	5 (3 - 7)	0.015	30	6 (4 - 9)	0.556
Non-invasive mechanical ventilation	383	60 (15.7%)	110	4 (3.6%)	161	30 (18.6%)	0.000	104	24 (23.1%)	0.000
Invasive mechanical ventilation	383	185 (48.3%)	110	67 (60.9%)	161	70 (43.5%)	0.005	104	46 (44.2%)	0.015
Time from ICU admission to MV (days)	185	2 (0 - 5)	67	1 (0 - 5)	70	1 (0 - 4)	0.802	46	4 (1 - 5)	0.034
MV duration (days)	185	13 (6 - 22)	67	15 (8 - 28)	70	13 (7 - 19)	0.175	46	8 (4 - 19)	0.029
**Antibiotics before mechanical ventilation**	**185**	**89 (48.1%)**	**67**	**35 (52.2%)**	**70**	**37 (52.9%)**	**0.942**	**46**	**15 (32.6%)**	**0.039**
**Catecholamines**	**383**	**145 (37.9%)**	**110**	**46 (41.8%)**	**161**	**57 (35.4%)**	**0.285**	**104**	**41 (39.4%)**	**0.721**
Time from ICU admission to catecholamines	145	2 (0 - 7)	46	1 (0 - 9)	57	2 (0 - 6)	0.872	41	4 (1 - 6)	0.249
Catecholamines duration (days)	145	8 (3 - 17)	46	15 (4 - 25)	57	7 (3 - 13)	0.014	41	5 (2 - 12)	0.003
**Renal replacement therapy**	**383**	**59 (15.4%)**	**110**	**26 (23.6%)**	**161**	**21 (13%)**	**0.024**	**104**	**11 (10.6%)**	**0.012**
Time from ICU admission to RRT	59	6 (1 - 14)	26	5 (0 - 14)	21	7 (3 - 15)	0.327	11	6 (2 - 10)	0.893
RRT duration (days)	59	10 (6 - 19)	26	8 (5 - 20)	21	10 (6 - 13)	0.723	11	14 (7 - 21)	0.424

Nb: Number of patients in whom the data was recorded, BMI: Body mass index, ICU: Intensive care unit, HFNCO: High Flow Nasal Cannula Oxygen, NIV: Non-Invasive Ventilation, MV: Invasive Mechanical Ventilation, RRT: Renal replacement therapy, *Period 2 vs. period 1, $period 3 vs. period 1

Table S2: This is a table. Comparison of survivors and non-survivors at hospital discharge

	Non-survivors		Survivors		
**Parameter**	**N**	**Result**	**N**	**Result**	**p**
Age (years)	14	69 (61 - 75)	24	60 (47 - 67)	0.000
Male gender	14	77 (53.8%)	24	131 (54.6%)	0.889
BMI (Kg/m^2)	12	29 (26 - 35)	22	30 (27 - 34)	0.323
Frailty	14	30 (21%)	24	12 (5%)	0.000
Simplified Acute Physiology Score	14	35 (29 - 44)	23	27 (22 - 33)	0.000
Occupancy rate during ICU stay (%)	14	94.6 (80.8-104)	24	93.1 (78.9-103.6)	0.495
**Comorbidities**					
Diabetes mellitus	14	70 (49%)	24	92 (38.3%)	0.042
Hypertension	14	104 (72.7%)	24	131 (54.6%)	0.000
Chronic respiratory failure	14	15 (10.5%)	24	13 (5.4%)	0.065
Chronic renal failure	14	23 (16.1%)	24	19 (7.9%)	0.013
Dialysis	14	7 (4.9%)	24	7 (2.9%)	0.318
Renal transplantation	14	2 (1.4%)	24	2 (0.8%)	0.599
Cardiac failure	14	15 (10.5%)	24	16 (6.7%)	0.185
Obesity	14	61 (42.7%)	24	127 (52.9%)	0.052
Pregnancy	66	1 (1.5%)	10	18 (16.5%)	0.002
Gestational age (weeks of gestation)	1	38 (38 - 38)	17	29 (28 - 35)	0.204
Sickle cell disease	14	2 (1.4%)	24	3 (1.3%)	0.901
Malignancy	14	8 (5.6%)	24	4 (1.7%)	0.033
Immunosuppression	14	16 (11.2%)	24	12 (5%)	0.024
**Time to hospitalization and ICU admission**					
Time between symptoms and hospitalization (days)	13	6 (3 - 8)	22	7 (4 - 9)	0.049
Time between hospitalization and ICU admission	14	1 (0 - 4)	24	1 (0 - 4)	0.532
ICU length of stay (days)	14	14 (7 - 23)	24	9 (6 - 15)	0.003
Hospital length of stay (days)	14	17 (9 - 27)	24	18 (13 - 29)	0.010
**Clinical manifestations**					
Number of organ failure	14	1 (1 - 2)	24	1 (1 - 1)	0.004
Hemodynamic failure	14	15 (10.5%)	24	9 (3.8%)	0.008
Respiratory failure	14	143 (100%)	24	240 (100%)	-
Neurologic failure	14	14 (9.8%)	24	11 (4.6%)	0.046
Hematologic failure	14	4 (2.8%)	24	3 (1.3%)	0.274
Renal failure	14	37 (25.9%)	24	33 (13.8%)	0.012
Liver failure	14	4 (2.8%)	24	6 (2.5%)	0.860
Infection at admission	14	6 (4.2%)	24	2 (0.8%)	0.026

Table S2: This is a table. Comparison of survivors and non-survivors at hospital discharge (Follow)

**Chest Computed-Tomography**	**143**	**125 (87.4%)**	**240**	**222 (92.5%)**	**0.099**
Alveolar condensations	125	44 (35.2%)	222	76 (34.2%)	0.856
Interstitial infiltrates	125	116 (92.8%)	222	215 (96.8%)	0.084
Pleural effusion	125	8 (6.4%)	222	21 (9.5%)	0.323
Posterior condensations	125	71 (56.8%)	222	110 (49.5%)	0.169
Contrast agent administration	125	84 (67.2%)	222	183 (82.4%)	0.001
Pulmonary embolism	84	12 (14.3%)	183	22 (12%)	0.606
Percentage of lesion	124	75 (50 - 75)	217	50 (50 - 75)	0.148
**Respiratory support at ICU admission**					
High flow nasal cannula oxygen	143	96 (67.1%)	240	185 (77.1%)	0.033
Non-invasive mechanical ventilation	143	55 (38.5%)	240	53 (22.1%)	0.001
Invasive mechanical ventilation	143	43 (30.1%)	240	53 (22.1%)	0.081
**Maximal respiratory support during hospitalization**					
High flow oxygen	143	18 (12.6%)	240	120 (50%)	0.000
HFNCO duration (days)	14	6 (3 - 8)	111	6 (4 - 8)	0.947
Non-invasive mechanical ventilation	143	16 (11.2%)	240	44 (18.3%)	0.063
Invasive mechanical ventilation	143	109 (76.2%)	240	76 (31.7%)	0.000
Time from ICU admission to MV (days)	109	3 (1 - 6)	76	1 (0 - 3)	0.000
MV duration (days)	109	13 (4 - 20)	76	14 (7 - 25)	0.123
**Antibiotics before mechanical ventilation**	**109**	**50 (45.9%)**	**76**	**39 (51.3%)**	**0.466**
**Catecholamines**	**143**	**96 (67.1%)**	**240**	**49 (20.4%)**	**0.000**
Time from ICU admission to catecholamines (days)	96	4 (0 - 9)	49	1 (0 - 3)	0.006
Catecholamines duration (days)	96	8 (3 - 17)	49	6 (3 - 16)	0.664
**Renal replacement therapy**	**143**	**42 (29.4%)**	**240**	**17 (7.1%)**	**0.000**
Time from ICU admission to RRT	42	8 (3 - 15)	17	1 (0 - 4)	0.006
RRT duration (days)	42	10 (4 - 15)	17	10 (6 - 21)	0.327
**ICU-AI**	**143**	**60 (42%)**	**240**	**37 (15.4%)**	**0.000**
Delay from ICU admission to ICU-AI (days)	60	9 (6 - 14)	37	9 (5 - 12)	0.549

Nb: Number of patients in whom the data was recorded, HFNCO: High Flow Nasal Cannula Oxygen, NIV: Non-Invasive Ventilation, MV: Invasive Mechanical Ventilation, RRT: Renal replacement therapy, ICU: Intensive care unit, AI: Acquired infection

Table S3: This is a table. Outcome of patients according to the study periods

	Total population		Period 1		Period 2			Period 3		
**Parameter**	**Nb**	**Result**	**Nb**	**Result**	**Nb**	**Result**	**p***	**Nb**	**Result**	**p$**
ICU length of stay (days)	383	10 (6 - 19)	110	13 (7 - 27)	161	9 (5 - 17)	0.003	104	10 (6 - 17)	0.027
Hospital length of stay (days)	383	18 (12 - 29)	110	21 (14 - 34)	161	17 (12 - 27)	0.027	104	15 (11 - 23)	0.001
ICU mortality	383	138 (36%)	110	33 (30%)	161	55 (34.2%)	0.473	104	49 (47.1%)	0.010
Limitation of life-sustaining treatment	143	54 (37.8%)	34	9 (26.5%)	59	27 (45.8%)	0.066	49	17 (34.7%)	0.427
Not to be intubated order	54	12 (22.2%)	9	1 (11.1%)	27	6 (22.2%)	0.652	17	5 (29.4%)	0.380
Mortality at day-30	383	115 (30%)	110	19 (17.3%)	161	50 (31.1%)	0.011	104	45 (43.3%)	0.000
Mortality at day-60	383	138 (36%)	110	32 (29.1%)	161	58 (36%)	0.234	104	47 (45.2%)	0.015
Hospital mortality	383	143 (37.3%)	110	34 (30.9%)	161	59 (36.6%)	0.329	104	49 (47.1%)	0.015
Causes of death :										
Septic shock	143	67 (46.9%)	34	21 (61.8%)	59	21 (35.6%)	0.015	49	25 (51%)	0.333
Severe hypoxemia	143	53 (37.1%)	34	8 (23.5%)	59	30 (50.8%)	0.010	49	15 (30.6%)	0.478
Pulmonary embolism	143	11 (7.7%)	34	4 (11.8%)	59	2 (3.4%)	0.187	49	5 (10.2%)	1
Others	143	12 (8.4%)	34	1 (2.9%)	59	6 (10.2%)	0.416	49	4 (8.2%)	0.644
ICU-Acquired infection	383	97 (25.3%)	110	34 (30.9%)	161	36 (22.4%)	0.114	104	27 (26%)	0.423
Time from admission to ICU-AI (days)	97	9 (6 - 14)	34	12 (8 - 18)	36	8 (5 - 12)	0.006	27	7 (5 - 14)	0.020

Nb: Number of patients in whom the data was recorded, ICU: Intensive care unit, AI: Acquired infection

*Period 2 *vs.* period 1, $period 3 *vs.* period 1
